# Independent and joint association of N-terminal pro-B-type natriuretic peptide and left ventricular mass index with heart failure risk in elderly diabetic patients with right ventricular pacing

**DOI:** 10.3389/fcvm.2022.941709

**Published:** 2022-07-22

**Authors:** Yu Yu, Hao Huang, Sijing Cheng, Yu Deng, Xi Liu, Min Gu, Xuhua Chen, Hongxia Niu, Chi Cai, Wei Hua

**Affiliations:** State Key Laboratory of Cardiovascular Disease, Cardiac Arrhythmia Center, Fuwai Hospital, National Center for Cardiovascular Diseases, Chinese Academy of Medical Sciences and Peking Union Medical College, Beijing, China

**Keywords:** NT-proBNP, left ventricular mass index, left ventricular hypertrophy, heart failure, elderly diabetes, right ventricular pacing

## Abstract

**Background:**

Elevated levels of N-terminal pro-B natriuretic peptide (NT-proBNP) and left ventricular hypertrophy (LVH) are independent risk factors for heart failure (HF). In addition, right ventricular pacing (RVP) is an effective treatment strategy for bradyarrhythmia, but long-term RVP is associated with HF. However, there is limited evidence on the independent and combined association of NT-proBNP and left ventricular mass index (LVMI) with HF risk in elderly diabetic patients with long-term RVP.

**Methods:**

Between January 2017 and January 2018, a total of 224 elderly diabetic patients with RVP at Fuwai Hospital were consecutively included in the study, with a 5-year follow-up period. The study endpoint was the first HF readmission during follow-up. This study aimed to explore the independent and joint relationship of NT-proBNP and LVMI with HF readmission in elderly diabetic patients with long-term RVP, using a multivariate Cox proportional hazards regression model.

**Results:**

A total of 224 (11.56%) elderly diabetic patients with RVP were included in the study. During the 5-year follow-up period, a total of 46 (20.54%) patients suffered HF readmission events. Multivariate Cox proportional hazards regression analysis showed that higher levels of NT-proBNP and LVMI were independent risk factors for HF readmission [NT-proBNP: hazard risk (HR) = 1.05, 95% confidence interval (CI): 1.01–1.10; LVMI: HR = 1.14, 95% CI: 1.02–1.27]. The optimal cut-off point of NT-proBNP was determined to be 330 pg/ml by receiver operating characteristic (ROC) curve analysis. Patients with NT-proBNP > 330 pg/ml and LVH had a higher risk of HF readmission compared to those with NT-proBNP ≤ 330 pg/ml and non-LVH (39.02% vs. 6.17%; HR = 7.72, 95% CI: 1.34–9.31, *P* < 0.001).

**Conclusion:**

In elderly diabetic patients with long-term RVP, NT-proBNP and LVMI were associated with the risk of HF readmission. Elevated NT-proBNP combined with LVH resulted in a significantly higher risk of HF readmission.

## Background

Approximately one million patients worldwide are currently undergoing pacemaker implantation annually (1), and more than 80% of them are over 65 years old (2). Studies have shown that about 25–35% of patients with pacemaker implantation have diabetes (3–5). Right ventricular pacing (RVP) is the conventional pacing strategy that is effective in treating atrioventricular block or bradyarrhythmia, including right ventricular apical and right ventricular septal pacing (2). Advanced age and diabetes are established risk factors for heart failure (HF) (6, 7), and long-term RVP is associated with a higher risk of HF (8). Considering the above factors, elderly diabetic patients experiencing long-term RVP are at a higher risk of HF. In order to reduce the risk of HF in such patients, we need to identify the risk factors associated with HF to address this clinical challenge better.

The N-terminal pro-B natriuretic peptide (NT-proBNP) is the main biological maker for the diagnosis of acute or chronic HF (9), and its predictive value for HF events has also been reported in some studies (10–12). However, the optimal cut-off value of NT-proBNP for predicting HF risk has not been reported in previous studies. Furthermore, the definition of higher NT-proBNP levels markedly varied across studies, dramatically limiting their clinical usefulness. It needs to be realized that HF is a complex clinical syndrome at the end stage of heart disease (13). Due to the high heterogeneity of the underlying disease, it is difficult to appropriately evaluate the long-term HF risk by relying on NT-proBNP alone (14).

Left ventricular mass index (LVMI), calculated by echocardiographic parameters and body surface area, is the primary marker for evaluating left ventricular hypertrophy (LVH) (15). Previous studies have demonstrated that the increased LVMI was an independent risk factor for HF in some specific populations (16–18). However, the different disease states have inconsistent effects on cardiac structure and function, and thus the results may not be generalizable to other patients. As mentioned above, although NT-proBNP is currently the most important biomarker for the diagnosis of HF, but it is susceptible to age, body mass index (BMI), diabetes, and renal function (19–22). Therefore, it is necessary to combine NT-proBNP and stable indicators to predict HF. Cardiac ultrasound has been widely used in cardiac patients, especially in patients with pacemaker implantation. LVMI has been shown in previous studies to be a valuable predictor of HF (17). Accordingly, the combination of NT-proBNP and LVMI may be able to improve the strength of the association with HF.

Since few studies have examined the relationship between NT-proBNP and LVMI on the risk of HF in elderly diabetic patients with long-term RVP. Therefore, this study aimed to investigate the relationship between NT-proBNP and LVMI on the risk of HF separately and to further explore whether elevated NT-proBNP levels and LVH have a higher cumulative risk of HF readmission in elderly diabetic patients with long-term RVP.

## Materials and methods

### Study participants

**FIGURE 1:**
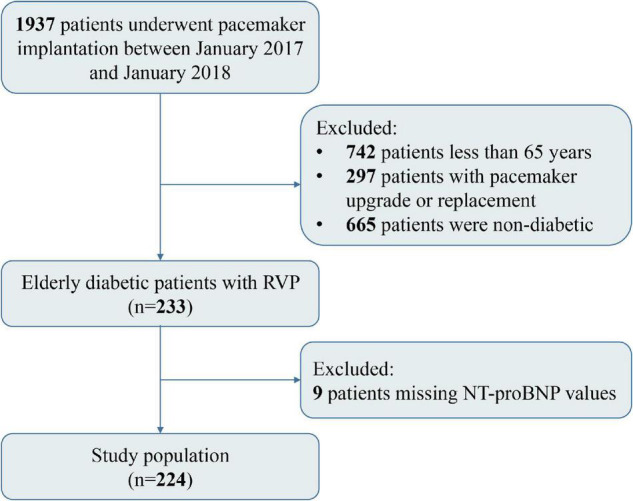
Flow chart of the study population.

This is a single-center, retrospective, observational cohort study. From January 2017 to January 2018, a total of 1937 patients underwent pacemaker implantation for sinus node dysfunction (SND) or atrioventricular block (AVB) at Fuwai Hospital (Chinese Academy of Medical Sciences, Beijing, China). The main inclusion criteria of this study: (1) Age ≥ 65 years old; (2) For diabetic patients undergoing pacemaker therapy for the first time, diabetes was defined as self-reported history of hypoglycemic drug use, and/or two or more times FBG ≥ 7.0 mmol/L during hospitalization. The exclusion criteria were as follows: (1) Pacemaker upgrading or replacement treatment on admission; (2) Missing baseline important information; (3) Lost to follow-up. We excluded 971 patients, of whom 297 patients underwent pacemaker upgrade or replacement, 665 patients were non-diabetic, and 9 patients had missing NT-proBNP values. Among them, 233 patients received RVP (including 83 patients with right ventricular septal pacing and 150 patients with right ventricular apical pacing). Finally, a total of 224 eligible patients were included in this study, and the selection process was presented in Figure 1. In addition, the non-RVP groups in the study include patients receiving His bundle pacing (HBP) or left bundle branch pacing (LBBP) due to bradyarrhythmia and those without pacing. The study was conducted in accordance with the Declaration of Helsinki and approved by the Ethics Committee of the Chinese Academy of Medical Sciences Fuwai Hospital. All patients signed a handwritten informed consent form before pacemaker implantation.

### Data collection

Patient demographics, medical history, echocardiographic parameters and laboratory tests were collected through the outpatient system of the hospital. Demographic characteristics including age, gender, body mass index (BMI), blood pressure, smoking history, and cardiac function. Medical history was mainly from admission records, operative records, and discharge diagnosis, including pacing lead location, postoperative ventricular pacing (VP) burden, SND, AVB, hypertension, coronary atherosclerotic disease (CAD), stroke, chronic kidney disease (CKD), left bundle branch block (LBBB) and cardiovascular drugs. Echocardiographic parameters included left ventricular ejection fraction (LVEF), left ventricular end-diastolic diameter (LVEDD), interventricular septum thickness (IVST), posterior wall thickness (PWT). Fasting blood samples were collected before pacemaker implantation. Laboratory tests included low-density lipoprotein cholesterol (LDL-C), fasting blood glucose (FBG), glycated hemoglobin A 1c (HbA1c), N-terminal pro-brain natriuretic peptide (NT-proBNP), and estimated glomerular filtration rate (eGFR).

### Calculation of left ventricular mass index and definition of left ventricular hypertrophy

The left ventricular mass (LVM) was calculated according to the Devereux formula (15), LVM (g) = 0.8 × 1.04 × ([IVST + LVEDD + PWT]^3^ – LVEDD^3^) + 0.6; Body surface area (BSA) was calculated according to Dubois method (23). LVMI (g/m^2^) = LVM/BSA. LVH was defined as LVMI > 115 g/m^2^ for male or LVMI > 95 g/m^2^ for female (24).

### Endpoints and follow-up

The endpoint of this study was the first HF readmission during follow-up, defined as an unplanned outpatient, or emergency department visit, or hospitalization of a patient due to signs and symptoms associated with HF, and use of diuretics during the visit, with significantly elevated NT-proBNP levels. The follow-up deadline was January 30, 2022. The length of follow-up was calculated from pacemaker implantation to the first readmission for HF or the follow-up deadline. The entire follow-up period lasted 5 years, with a median of 53 months of follow-up.

### Statistical analysis

Continuous variables were expressed as mean ± standard deviation (SD) or median (interquartile range), and categorical variables were expressed as percentages (%). Normality in the distribution of the data was checked through normality tests. Baseline characteristics of patients between groups were analyzed by one-way ANOVA (continuous variables) or chi-square test (categorical variables). Univariate and Cox proportional hazards models were used to identify risk factors associated with HF readmission by calculating HR and 95% CI. Cox proportional hazards models were used to assess the joint relationship of NT-proBNP and LVH with the risk of HF readmission. Kaplan Meier curves (log-rank test) were used to demonstrate the HF risk of each group during the follow-up period, and the log-rank test revealed differences between groups. The optimal cut-point for NT-proBNP was determined by receiver operating characteristic (ROC) curve analysis.

A 2-tailed *P* < 0.05 was considered statistically significant. All statistical analysis results were obtained through the statistical packages R, Empower (R) (X&Y Solutions, Inc., Boston, MA), and SPSS (IBM SPSS 23.0, SPSS Inc.).

## Results

### Baseline characteristics

A total of 224 elderly diabetic patients with RVP were included in this study, with a mean age of 75.76 ± 6.06 years, a male proportion of 51.79%, a mean LVEF of 62.01 ± 4.68%. The distribution of NT-proBNP was non-normal, with a median (Q1-Q3) of 344.65 (158.15–921.92) pg/ml (Supplementary Figure 1). The distribution of LVMI was non-normal, with a mean (SD) of 95.36 (22.40) g/m^2^ g/m^2^ (Supplementary Figure 2). The ROC curve determined the optimal NT-proBNP cut-off value as 330 pg/ml (Supplementary Figure 3 and Supplementary Table 1). The baseline characteristics of patients were divided into two groups according to the NT-proBNP cut point (NT-proBNP ≤ 330 pg/ml, NT-proBNP > 330 pg/ml) and are shown in Table 1. Patients with NT-proBNP > 330 pg/ml had higher age, BMI, and LVMI values and a higher proportion of VP burden; however, LVEF, LDL-C, and eGFR values were lower than the NT-proBNP ≤ 330 pg/ml group. In addition, the baseline characteristics of patients were also divided into two groups according to LVH (non-LVH, LVH). The LVH group had higher NT-proBNP values and a higher proportion of hypertension and cardiovascular medications, lower LVEF values, and a lower proportion of males (Table 2).

**TABLE 1:** Baseline characteristics of study participants stratified by NT-proBNP.

Characteristics	Total population	NT-proBNP ≤ 330 pg/mL	NT-proBNP > 330 pg/mL	*P*-value
	(*N* = 224)	(*N* = 110)	(*N* = 114)	
Age, years	75.65 ± 6.03	74.01 ± 5.29	77.24 ± 6.29	**<0.001**
Male, n (%)	116 (51.79%)	59 (53.64%)	57 (50.00%)	0.586
BMI, kg/m^2^	25.11 ± 3.58	25.50 ± 3.20	24.72 ± 3.89	**0.026**
Current smoker, n (%)	76 (34.23%)	37 (33.94%)	39 (34.51%)	0.929
Cardiac function, n (%)				0.33
NYHA I-II	220 (98.21%)	109 (99.09%)	111 (97.37%)	
NYHA III-IV	4 (1.79%)	1 (0.91%)	3 (2.63%)	
Lead location, n (%)				0.824
RVS	79 (35.27%)	38 (34.55%)	41 (35.96%)	
RVA	145 (64.73%)	72 (65.45%)	73 (64.04%)	
VP burden,%	42.03 ± 41.66	36.82 ± 41.52	47.80 ± 41.29	**0.014**
SBP, mmHg	140.87 ± 18.63	140.25 ± 16.15	141.46 ± 20.80	0.625
DBP, mmHg	69.00 ± 10.71	69.39 ± 11.69	68.61 ± 9.70	0.588
LVEF,%	62.01 ± 4.68	62.87 ± 3.59	61.18 ± 5.41	**0.006**
LVMI, g/m^2^	95.36 ± 22.40	92.78 ± 20.27	97.84 ± 24.10	**0.031**
SND, n (%)	148 (66.07%)	78 (70.91%)	70 (61.40%)	0.133
AVB, n (%)	77 (34.38%)	39 (35.45%)	38 (33.33%)	0.738
Hypertension, n (%)	192 (85.71%)	95 (86.36%)	97 (85.09%)	0.785
CAD, n (%)	123 (54.91%)	56 (50.91%)	67 (58.77%)	0.237
Stroke, n (%)	57 (25.45%)	23 (20.91%)	34 (29.82%)	0.126
CKD, n (%)	31 (13.90%)	5 (4.59%)	26 (22.81%)	
LBBB, n (%)	14 (6.25%)	6 (5.45%)	8 (7.02%)	0.629
Cardiovascular drugs^†^, n (%)	210 (93.75%)	101 (91.82%)	109 (95.61%)	0.241
LDL-C, mmol/L	2.27 ± 0.85	2.43 ± 0.95	2.11 ± 0.73	**0.005**
FBG, mmol/L	7.29 ± 2.52	7.14 ± 2.31	7.43 ± 2.72	0.395
HbA1c level,%	7.12 ± 1.18	7.16 ± 1.23	7.08 ± 1.13	0.617
NT-proBNP, Median (Q1-Q3), pg/mL	344.65 (158.15–921.92)	156.65 (89.20–217.53)	912.70 (545.60–1312.40)	**<0.001**
eGFR, mL/min/1.73 m^2^	67.04 ± 14.77	71.52 ± 10.70	62.71 ± 16.77	**<0.001**

Data are presented as mean ± standard deviation (SD), or median (interquartile range) or number (%). P-values in bold are < 0.05.

^†^Included antihypertensive drugs, statins, and anti-thrombotic agents.

NT-proBNP, N-terminal pro-brain natriuretic peptide; BMI, body mass index; NYHA, New York Heart Association; RVS, right ventricular septum; RVA, right ventricular apex; VP, ventricular pacing; SBP, systolic blood pressure; DBP, diastolic blood pressure; LVEF, left ventricular ejection fraction; LVMI, left ventricular mass index; SND, sinus node dysfunction; AVB, atrioventricular block; CAD, coronary atherosclerotic disease; CKD, chronic kidney disease; LBBB, left bundle branch block; LDL-C, low-density lipoprotein cholesterol; FBG, fasting blood glucose; HbA1c, glycated hemoglobin; eGFR, estimated glomerular filtration rate.

**TABLE 2:** Baseline characteristics of study participants stratified by LVH.

Characteristics	Total population(*N* = 224)	Non-LVH (*N* = 154)	LVH (*N* = 70)	*P*-value
Age, years	75.65 ± 6.03	75.84 ± 5.78	75.24 ± 6.57	0.495
Male, n (%)	116 (51.79%)	88 (57.14%)	28 (40.00%)	**0.017**
BMI, kg/m^2^	25.11 ± 3.58	24.99 ± 3.55	25.36 ± 3.67	0.47
Current smoker, n (%)	76 (34.23%)	51 (33.55%)	25 (35.71%)	0.752
Cardiac function, n (%)				0.786
NYHA I-II	220 (98.21%)	151 (98.05%)	69 (98.57%)	
NYHA III-IV	4 (1.79%)	3 (1.95%)	1 (1.43%)	
Lead location, n (%)				0.266
RVS	79 (35.27%)	58 (37.66%)	21 (30.00%)	
RVA	145 (64.73%)	96 (62.34%)	49 (70.00%)	
VP burden,%	42.03 ± 41.66	41.15 ± 40.72	43.85 ± 43.83	0.687
SBP, mmHg	140.87 ± 18.63	140.36 ± 18.55	141.97 ± 18.91	0.551
DBP, mmHg	69.00 ± 10.71	69.32 ± 11.06	68.29 ± 9.92	0.505
LVEF,%	62.01 ± 4.68	62.44 ± 4.53	61.06 ± 4.89	**0.04**
LVMI, g/m^2^	95.36 ± 22.40	84.01 ± 12.72	120.31 ± 18.53	**<0.001**
SND, n (%)	148 (66.07%)	102 (66.23%)	46 (65.71%)	0.939
AVB, n (%)	77 (34.38%)	55 (35.71%)	22 (31.43%)	0.531
Hypertension, n (%)	192 (85.71%)	126 (81.82%)	66 (94.29%)	**0.013**
CAD, n (%)	123 (54.91%)	83 (53.90%)	40 (57.14%)	0.651
Stroke, n (%)	57 (25.45%)	39 (25.32%)	18 (25.71%)	0.951
CKD, n (%)	31 (13.90%)	17 (11.11%)	14 (20.00%)	0.075
LBBB, n (%)	14 (6.25%)	9 (5.84%)	5 (7.14%)	0.71
Cardiovascular drugs^†^, n (%)	210 (93.75%)	140 (90.91%)	70 (100.00%)	**0.009**
LDL-C, mmol/L	2.27 ± 0.85	2.25 ± 0.77	2.33 ± 1.02	0.517
FBG, mmol/L	7.29 ± 2.52	7.39 ± 2.53	7.06 ± 2.52	0.37
HbA1c level,%	7.12 ± 1.18	7.16 ± 1.23	7.03 ± 1.04	0.425
NT-proBNP, Median (Q1-Q3), pg/mL	344.65 (158.15–921.92)	303.70 (131.53–814.98)	406.55 (197.65–1079.75)	**0.044**
eGFR, mL/min/1.73 m^2^	67.04 ± 14.77	67.63 ± 13.89	65.72 ± 16.56	0.371

Data are presented as mean ± standard deviation (SD), or median (interquartile range) or number (%). P-values in bold are < 0.05.

^†^Included antihypertensive drugs, statins, and anti-thrombotic agents.

LVH, left ventricular hypertrophy; NT-proBNP, N-terminal pro-brain natriuretic peptide; BMI, body mass index; NYHA, New York Heart Association; RVS, right ventricular septum; RVA, right ventricular apex; VP, ventricular pacing; SBP, systolic blood pressure; DBP, diastolic blood pressure; LVEF, left ventricular ejection fraction; LVMI, left ventricular mass index; SND, sinus node dysfunction; AVB, atrioventricular block; CAD, coronary atherosclerotic disease; CKD, chronic kidney disease; LBBB, left bundle branch block; LDL-C, low-density lipoprotein cholesterol; FBG, fasting blood glucose; HbA1c, glycated hemoglobin; eGFR, estimated glomerular filtration rate.

### The independent association of N-terminal pro-B natriuretic peptide and left ventricular mass index on heart failure readmission

Table 3 shows the results of univariate and multivariate Cox regression analyses of HF readmission. In the univariate analysis model, the age, LVMI, and NT-proBNP were positively associated with the incidence of HF readmission, whereas the LVEF and eGFR were inversely associated with HF readmission. In the multivariate Cox regression model, LVMI and NT-proBNP were positively associated with the risk of HF readmission, respectively (NT-proBNP: HR = 1.05, 95%CI 1.01–1.10; LVMI: HR = 1.14, 95% CI: 1.02–1.27).

**TABLE 3:** Univariate and multivariate cox regression analyses for the association between variables and HF readmission.

Variables	Univariate analysis		Multivariate analysis	
	HR (95%CI)	*P*-value	HR (95%CI)	*P*-value
Age	1.06 (1.01, 1.11)	**0.018**		
Female	0.87 (0.49, 1.55)	0.639		
BMI	0.99 (0.91, 1.07)	0.767		
Current smoker	1.18 (0.65, 2.15)	0.585		
Cardiac function (NYHA III-IV)	2.49 (0.60, 10.28)	0.207		
SBP	1.00 (0.98, 1.01)	0.936		
LVEF	0.91 (0.87, 0.95)	**<0.001**		
LVMI*	1.15 (1.03, 1.29)	**0.015**	**1.14 (1.02, 1.27)**	**0.030**
CAD	1.33 (0.74, 2.40)	0.337		
LBBB	1.85 (0.73, 4.69)	0.192		
LDL-C	0.96 (0.68, 1.35)	0.820		
HbA1c	1.21 (0.98, 1.50)	0.079		
NT-proBNP^†^	1.05 (1.03, 1.08)	**<0.001**	**1.05 (1.01, 1.10)**	**0.047**
eGFR	0.96 (0.94, 0.97)	**<0.001**	**0.96 (0.93, 0.98)**	**0.001**

P-values in bold are < 0.05.

*Indicates per 1-unit changed in LVMI is 10 g/m^2^.

^†^Indicates per 1-unit changed in NT-proBNP is 100 pg/ml.

HF, heart failure; BMI, body mass index; NYHA, New York Heart Association; SBP, systolic blood pressure; LVEF, left ventricular ejection fraction; LVMI, left ventricular mass index; LBBB, left bundle branch block; LDL-C, low-density lipoprotein cholesterol; HbA1c, glycated hemoglobin; NT-proBNP, N-terminal pro-brain natriuretic peptide; eGFR, estimated glomerular filtration rate, HR, hazards ratio; CI, Confidence interval.

### The joint relationship of N-terminal pro-B natriuretic peptide and left ventricular hypertrophy with heart failure risk

**FIGURE 2:**
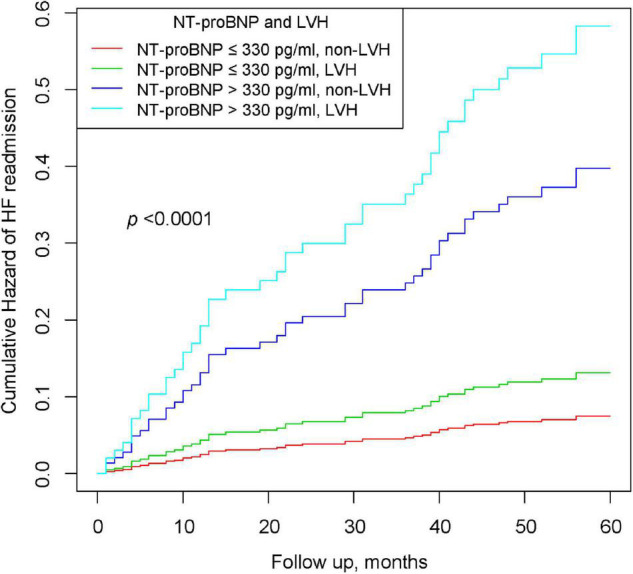
Kaplan–Meier curves of cumulative hazards of HF readmission stratified by NT-proBNP and LVH.

In Table 4, patients with preserved NT-proBNP (≤330 pg/ml) and LVMI (non-LVH) were used as a reference, and those with NT-proBNP (≤330 pg/ml) and increased LVMI (LVH) had a higher risk of HF (HR 1.67, 95% CI: 0.39–5.18). Similarly, patients with non-LVH and elevated NT-proBNP (>330 pg/ml) experienced a higher risk of HF (HR 4.71, 95% CI: 1.68–9.19). More importantly, patients with elevated NT-proBNP (>330 pg/ml) and LVH had a significantly higher cumulative risk of HF. The *P* for trend <0.001 indicated that the risk of HF readmission was progressively increasing among the four groups. Figure 2 shows the K-M curves for the cumulative risk of HF readmission in the four groups, with statistical differences between the groups (log-rank *P* < 0.001). The risk of HF readmission was highest in the group with NT-proBNP > 330 pg/ml and LVH throughout the follow-up period.

**TABLE 4:** Combined associations of NT-proBNP (≤330 pg/ml, >330 pg/ml) and LVH on the incidence of HF readmission.

Combined variables	Events, %	HF readmission, HR (95% CI)		
		Model 1	Model 2	Model 3
NT-proBNP ≤ 330 pg/ml, non-LVH	5 (6.17%)	1	1	1
NT-proBNP ≤ 330 pg/ml, LVH	3 (10.34%)	1.69 (0.40, 5.07)	1.76 (0.42, 5.41)	1.67 (0.39, 5.18)
NT-proBNP > 330 pg/ml, non-LVH	22 (30.14%)	5.66 (2.14, 9.94)	5.30 (1.97, 9.24)	4.71 (1.68, 9.19)
NT-proBNP > 330 pg/ml, LVH	16 (39.02%)	7.78 (2.85, 11.27)	7.76 (2.80, 11.50)	7.72 (2.79, 11.90)
*P* for trend		<0.001	<0.001	<0.001

Model 1, adjusted for none; Model 2, adjusted for age, sex; Model 3, adjusted for age, sex; BMI, current smoker, NYHA, SBP, LVEF, CAD, LDL-C, eGFR.

NT-proBNP, N-terminal pro-brain natriuretic peptide; LVH, left ventricular hypertrophy; HF, heart failure; BMI, body mass index; NYHA, New York Heart Association; LVEF, left ventricular ejection fraction; CAD, coronary atherosclerotic disease; LDL-C, low-density lipoprotein cholesterol; eGFR, estimated glomerular filtration rate; HR, hazards ratio; CI, confidence interval.

## Discussion

This study is a real-world, observational cohort of 224 elderly diabetic patients with RVP followed up for 5 years. The results suggested that NT-proBNP and LVH were independent risk factors for HF readmission, respectively. Furthermore, patients with higher levels of NT-proBNP and LVH have a cumulative risk of HF readmission. Therefore, the findings suggest that we should monitor both early NT-proBNP and LVMI levels in order to better reduce the onset of HF.

Compared with BNP, NT-proBNP has a longer plasma half-life and better stability, so it is more suitable for clinical diagnosis and prediction of HF (25). Previous studies reported that early higher NT-proBNP levels were associated with long-term HF risk. Bettencourt et al. (10) enrolled 182 patients with chronic HF and found that a >30% increase in NT-proBNP was associated with a higher risk of HF at 6 months (HR: 6.64, 95% CI: 3.60–12.23). Verdiani et al. (11) enrolled 120 patients with acute HF and showed that a decrease of <30% in NT-proBNP during hospitalization was associated with a higher risk of HF at 6 months (HR: 2.04, 95% CI: 1.02–4.08). O’Brien et al. (12) included 96 patients with acute HF and revealed that NT-proBNP was positively associated with the risk of HF at 1 year (OR: 15.30, 95% CI: 1.4–16.9). Although the above studies showed that elevated NT-proBNP levels were associated with an increased risk of HF, these studies had a short follow-up period and did not determine an optimal cut-off value for NT-proBNP. Our results show that higher NT-proBNP levels are associated with long-term HF risk, which has not been reported previously.

The mechanisms underlying the relationship between NT-proBNP and HF have been well studied. NT-proBNP is mainly synthesized by cardiomyocytes, is secreted in large amounts in the presence of passive ventricular dilation and volume overload, and has diuretic and vasodilatory effects (26). In pre-HF stage, an increase in ventricular volume and pressure load will enhance the ventricular wall tension, leading to activation of the natriuretic peptide system and an increase in plasma NT-proBNP concentration (27). In addition, advanced age and renal insufficiency can lead to poor ventricular compliance and increased ventricular filling pressure, resulting in elevated NT-proBNP level (28, 29). Notably, our results showed a positive correlation between NT-proBNP and VP burden, suggesting that higher VP burden might be a risk factor for HF. RVP generates retrograde conduction of electrical impulses that will lead to asynchronous activation of the ventricles (30). Thus, long-term RVP causes cardiac remodeling and ultimately increases the risk of HF. This result was produced by the specific population of this study, so it is a novel finding of our study.

The definition of LVMI and its use as a criterion for the diagnosis of LVH was proposed by Devereux and Reichek (15) in 1977, and then several studies have found an independent positive association between LVMI and HF in cardiovascular population (16–18). These previous studies were largely limited to Western populations; however, there are ethnic differences in body composition and risk of heart disease between Chinese and Western populations due to ethnic, geographic, dietary, and other differences (31, 32). Furthermore, different underlying diseases can cause various degrees of damage to cardiac structure and function (33), so the relationship between LVMI and HF still needs to be reexamined in other populations. Our results suggest that higher LVMI levels are an independent risk factor for HF in elderly diabetic patients with RVP, which is similar to the previous findings. The possible mechanisms can be explained as follows. Sustained ventricular hypertrophy will induce inadequate energy supply to cardiac myocytes, reduce ventricular muscle compliance, and ultimately the onset of HF (34).

The further result revealed that higher NT-proBNP and LVH have a cumulative risk for HF readmission, and this result was in agreement with expectations. LVH reflects structural abnormalities of the heart and triggers an increase in ventricular filling pressure and ventricular wall pressure, thereby stimulating excessive NT-proBNP secretion by cardiac myocytes (35, 36). In addition, animal studies suggest that long-term RVP may increase LV mass, and thus cause an increase in LV filling, a reduction in cardiac output, and abnormalities in myocardial metabolism (37). These factors together contribute to the decline in cardiac function, but further studies are needed to elucidate the specific biological mechanisms underlying the development of HF in this population.

Interestingly, almost all patients in this study had a normal baseline LVEF (>50%). This phenomenon has important implications for this study: first, most patients did not experience a severe cardiac function decline at baseline; second, this is because elderly diabetic patients are more likely to develop HF with preserved ejection fraction (HFpEF) (38). Therefore, the predictive value of LVEF is limited for such patients, and the combination of LVEF and NT-proBNP failed to be a meaningful risk factor for HF. However, this dilemma is well avoided in our study that the elevated NT-proBNP and LVH were significantly associated with a higher risk of HF in patients with HFpEF. The diagnosis and treatment of HFpEF have long been a challenge for clinicians (39). The ACEI/ARB, beta-blockers, and MRA, widely used in treating patients with HFrEF, have failed to be proven by randomized clinical trials to improve the clinical prognosis of patients with HFpEF (40). Subsequently, the PARAGON-HF study also failed to confirm that ARNI improves the prognosis of patients with HFpEF (41). To date, only the EMPEROR-Preserved trial has shown that SGLT2 inhibitors improve the clinical prognosis of patients with HFpEF (42), which provides new insights into the medical treatment of patients with HFpEF. The above findings suggest that the causes of HFpEF are not fully elucidated, and it is important to explore the risk factors associated with HFpEF patients to improve their prognosis. Based on the findings of this study, clinicians should focus not only on the changes in NT-proBNP levels but also on the effects of left ventricular hypertrophy (LVH) on cardiac function. LVH would result in elevated LV filling pressures by impairing diastolic function, which is one of the pathophysiological bases of HFpEF (40). Our study further highlights the important role of LVH in the pathogenesis of HFpEF, and future clinical treatments focusing on delaying or even reversing LVH may help improve the clinical prognosis of patients with HFpEF.

In addition, at the beginning of the study, we tried to explore the joint relationship of NT-proBNP and LVMI with heart failure (HF) in elderly diabetic patients with RV pacing and non-RV pacing. However, for the reasons that our center currently lacks a sufficient number of patients with non-RV pacing. A recent Danish national study investigated the difference in HF hospitalization between patients with and without RVP, and found that patients with RVP experienced a nearly 6-fold increased risk of HF compared to those without RVP (HR 5.98, 95% CI: 5.19–6.9, *P* < 0.001) (43). In HF patients caused by long-term, high-burden RVP who develop significant HF symptoms and LVEF ≤ 35% despite optimal medical therapy, the 2021 ESC guidelines recommend upgrading to CRT to improve the prognosis of such patients (2). Notably, with the increasing evidence on physiological pacing, several studies have shown that HBP and LBBP can be effective treatment strategies to improve the prognosis of HF patients (44, 45). Although these results are from observational studies, the evidence suggests that physiological pacing is a promising strategy for treating HF and deserves further promotion and application in the clinical setting.

As the first study to explore the joint association of NT-proBNP and LVMI with heart failure, the main clinical implications are as follows. First, our findings reveal a possible accumulative contribution of NT-proBNP and LVMI to the onset and progression of HF. This finding may help provide a direction for the following basic research to explore the mechanism of HF. Second, the findings suggest that clinicians should pay attention to both NT-proBNP and LVMI; the results may help promote the application of LVMI in clinical practice. The combination of NT-proBNP and LVMI is more robust and has a stronger association with HF than NT-proBNP alone. The application of this joint index in the clinic may help to identify high-risk patients as early as possible and thus provide assistance in preventing the development of HF.

The strength of this study is that we investigated the independent relationship of NT-proBNP and LVMI with HF readmission, and further ascertained that higher levels of NT-proBNP and LVH have a cumulative risk of HF readmission. Some potential limitations should be mentioned. Firstly, the patients in this study were from a single center and sample size was relatively small. Therefore, the results of this study need to be verified by future large-sample, multi-center studies. Secondly, this study only collected baseline information and did not collect data during the follow-up period, so it failed to analyze the dynamic changes of risk factors. However, previous studies reported that LVMI values did not change significantly in the short term (46), so the combination of NT-proBNP and LVMI is a stable indicator.

## Conclusion

The results suggested that NT-proBNP and LVH were independent risk factors for HF readmission. Furthermore, elevated NT-proBNP levels and LVH have a higher cumulative risk for HF readmission in elderly diabetic patients with long-term RVP. Early investigation of NT-proBNP levels and assessment of LVH status may be beneficial for cardiovascular risk stratification and reduction of HF readmission risk in elderly diabetic patients with long-term RVP.

## Data availability statement

The raw data supporting the conclusions of this article will be made available by the authors, without undue reservation.

## Ethics statement

The studies involving human participants were reviewed and approved by Ethics Committee of the Chinese Academy of Medical Sciences Fuwai Hospital. The patients/participants provided their written informed consent to participate in this study.

## Author contributions

YY and WH participated in the study design and interpreted data, drafted, and edited the report. YY, HH, SC, YD, XL, MG, HN, XC, and CC participated in data collection. YY and HH performed the statistical analysis. All authors read and approved the final manuscript.

## Conflict of interest

The authors declare that the research was conducted in the absence of any commercial or financial relationships that could be construed as a potential conflict of interest.

## Publisher’s note

All claims expressed in this article are solely those of the authors and do not necessarily represent those of their affiliated organizations, or those of the publisher, the editors and the reviewers. Any product that may be evaluated in this article, or claim that may be made by its manufacturer, is not guaranteed or endorsed by the publisher.

## Abbreviations

HF: heart failure
LVH: left ventricular hypertrophy
NT-proBNP: N-terminal pro-brain natriuretic peptide
BMI: body mass index
NYHA: New York Heart Association
RVS: right ventricular septum
RVA: right ventricular apex
VP: ventricular pacing
SBP: systolic blood pressure
DBP: diastolic blood pressure
LVEF: left ventricular ejection fraction
LVMI: left ventricular mass index
SND: sinus node dysfunction
AVB: atrioventricular block
CAD: coronary atherosclerotic disease
CKD: chronic kidney disease
LBBB: left bundle branch block
LDL-C: low-density lipoprotein cholesterol
FBG: fasting blood glucose
HbA1c: glycated hemoglobin
eGFR: estimated glomerular filtration rate
HR: hazard ratio
CI: confidence interval.
