# Supplementary material for: Independent and joint association of N-terminal pro-B-type natriuretic peptide and left ventricular mass index with heart failure risk in elderly diabetic patients with right ventricular pacing
Source: Front Cardiovasc Med. 2022 Jul 22;9:941709. doi: 10.3389/fcvm.2022.941709 (PMC9354452; doi:10.3389/fcvm.2022.941709)
Supplement: Supplementary file 1 [file Data_Sheet_1.docx]

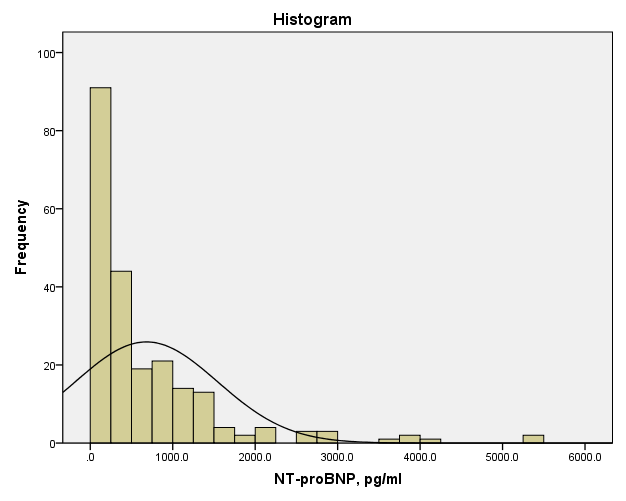


**Fig. S1** Distribution of NT-proBNP

Median (Q1-Q3) = 344.65 (158.15-921.92) pg/ml


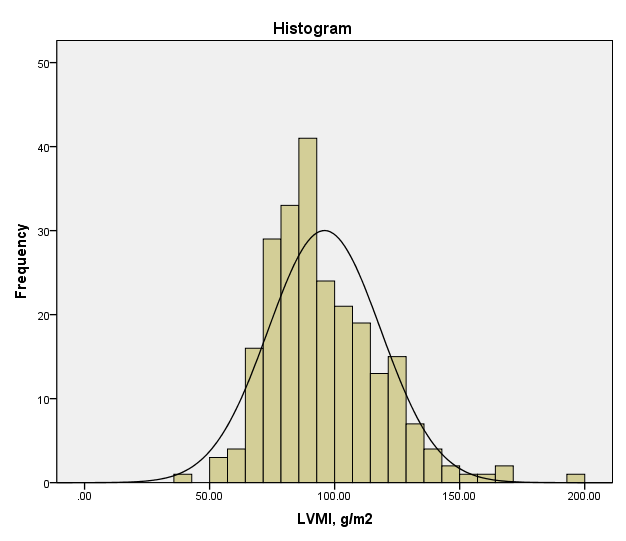


**Fig. S2** Distribution of LVMI

Mean (SD) = 95.36 (22.40) g/m2


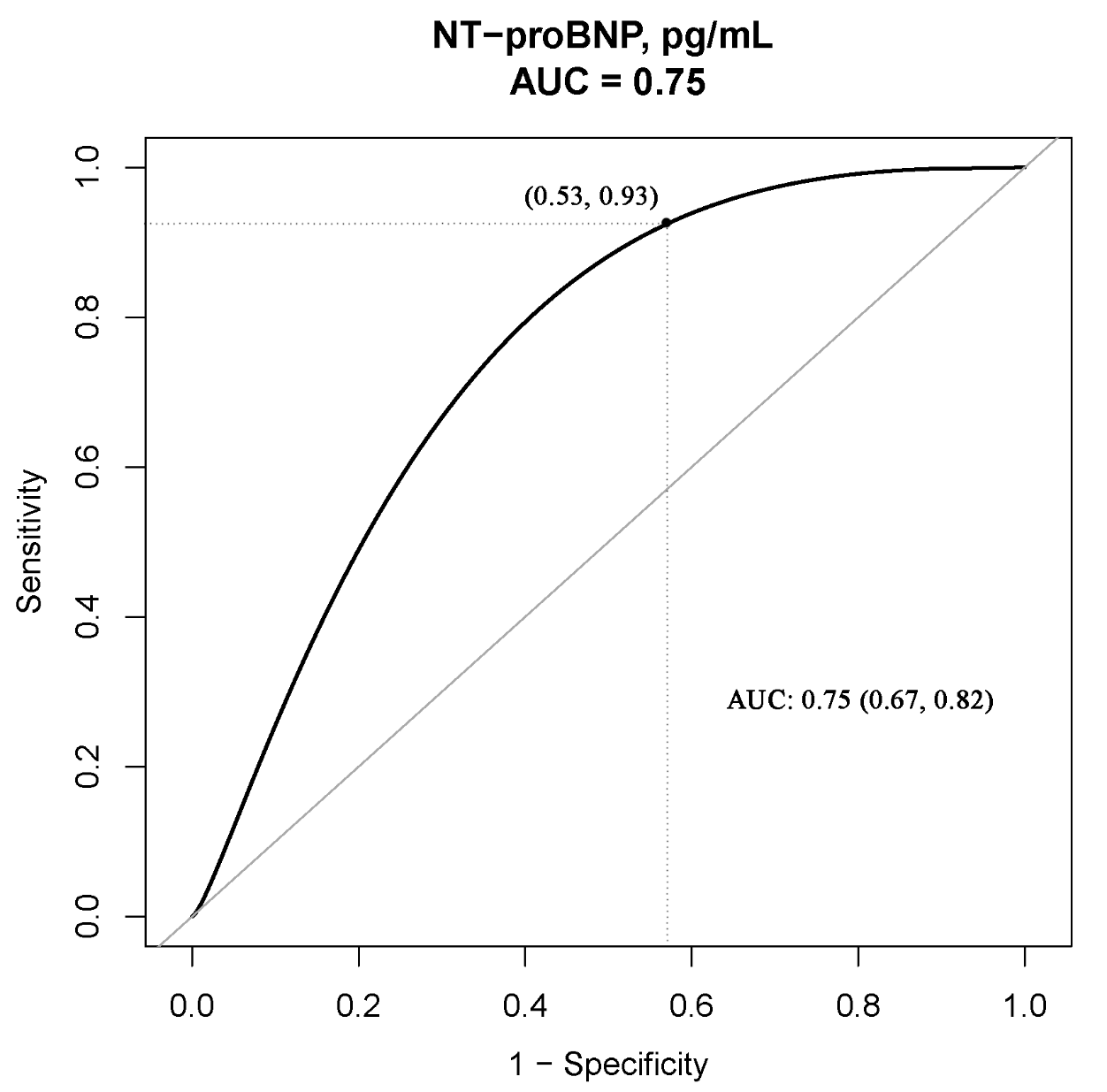


**Fig. S3** Receiver operating characteristic (ROC) curve of NT-proBNP

| **Table S1.** Best threshold analysis of ROC analysis | | | | | | |
| --- | --- | --- | --- | --- | --- | --- |
| Test | Best threshold | ROC area (AUC) | 95%CI low | 95%CI upp | Specificity | Sensitivity |
| NT-proBNP, pg/mL | 330.00 | 0.75 | 0.67 | 0.82 | 0.47 | 0.93 |
